# Astrocytes induce desynchronization and reduce predictability in neuron–astrocyte networks cultured on microelectrode arrays

**DOI:** 10.1098/rsos.240839

**Published:** 2024-10-30

**Authors:** Barbara Genocchi, Annika Ahtiainen, Annika Niemi, Michael T. Barros, Jarno M. A. Tanskanen, Kerstin Lenk, Jari Hyttinen, Narayan Puthanmadam Subramaniyam

**Affiliations:** ^1^ Faculty of Medicine and Health Technology, Tampere University, Tampere, Finland; ^2^ School of Computer Science and Electronic Engineering, University of Essex, Colchester, Essex, UK; ^3^ Institute of Neural Engineering, Graz University of Technology, Graz, Austria; ^4^ BioTechMed, Graz, Austria

**Keywords:** neuronal networks, astrocytes, microelectrode arrays, signal analysis, ionic buffering

## Abstract

In this article, we aim to study how astrocytes control electrophysiological activity during neuronal network formation. We used a combination of spike/burst analysis, classification of spike waveforms based on various signal properties and tools from information theory to demonstrate how astrocytes modulate the electrical activity of neurons using microelectrode array (MEA) signals. We cultured rat primary cortical neurons and astrocytes on 60-electrode MEAs with different neuron/astrocyte ratios ranging from ‘pure’ neuronal cultures to co-cultures containing 50% neurons and 50% astrocytes. Our results show that astrocytes desynchronize the network and reduce predictability in the signals and affect the repolarization phase of the action potentials. Our work highlights that it is crucial to go beyond standard MEA analysis to assess how astrocytes control neuronal cultures and investigate any dysfunction that could potentially result in neuronal hyperactivity.

## 1. Introduction

Astrocytes, a type of glial cells, are localized in the central nervous system (CNS) and are interconnected with neurons in the so-called tripartite synapses [1]. Astrocytes play important roles in the CNS, from initial development to ongoing homeostasis. They actively promote synapse formation, maturation and elimination [2]. Furthermore, astrocytes provide metabolic support to the neurons [3] and maintain homeostasis by removing excessive ions and neurotransmitters from the extracellular space [4–6]. Astrocytes’ ability to remove K^+^ ions from the extracellular space is crucial for regulating neuronal activity [7]. Astrocytes also modulate neuronal activity through the release of both excitatory and inhibitory neurotransmitters [8].

Microelectrode array (MEA) measurements have been traditionally used to record neuronal activity [9], including action potential spikes. Measurements in MEAs have the advantages of being non-invasive, real-time and network-wide; they also make it possible to study cultures over extended time periods by tracking network activity and its changes [10–12].

For neurons in resting state, Na^+^ ions are more concentrated in the extracellular space while K^+^ ions are more concentrated in the intracellular space. This results in a negative membrane potential since there are more positive charges in the extracellular space. When a sufficient stimulus reaches the membrane, Na^+^ channels open, allowing Na^+^ ions to flow into the cell and reversing the membrane potential to a positive value [13]. During the subsequent repolarization, K^+^ channels open and extrude K^+^ ions from the cell, leading to an excess of positive charges in the extracellular space and a negative membrane potential. Understanding how ionic clearance impacts neuronal signalling is crucial because astrocytic dysfunctions can cause, for example, epileptic seizures [14,15]. Even though astrocytes highly impact neuronal activity, studies using co-cultures of neurons and astrocytes with defined cell ratios on MEAs are needed to characterize the full impact of astrocytes in neuronal information regulation. The majority of the studies use co-cultures to improve the survival rate or for drug testing, rather than studying the actual effects of astrocytes on neuronal activity [16–22]. In a previous work, we studied how astrocytes balanced hyperactivity induced by drugs like gabazine and 4-aminopyridine (4-AP) [17]. We cultured four co-cultures with different fixed ratios of added astrocytes, and we found that co-cultures with a higher ratio of astrocytes effectively counteracted 4-AP-induced hyperactivity.

Astrocytes have been shown to affect network synchronicity in both *in vitro* and *in vivo* studies, which is also supported by computational models of neuron–astrocyte networks [23,24]. Synchronicity in MEA recordings has been traditionally assessed from the variability of inter-spike intervals (ISIs) and inter-burst intervals (IBIs). However, with these techniques there are two main concerns: (i) different burst detection algorithms lead to different results, making the comparison of results difficult [25]; and (ii) in the transition from the raw signals to the binary spike trains, much of the signal information is lost, e.g. peak amplitude, peak duration and all the information buried in the background noise, including the action potential spikes below the spike detection threshold.

Complexity–entropy (C-E) causality planes have been used to discriminate chaotic signals from more complex patterned signals in several fields. This complexity metric describes the physical structure of the signal [26], whereas the entropy defines the level of regularity in the signal [27]. In neuroscience, these measures have been most commonly used in electroencephalography (EEG) signal analysis [28–32].

In the presented work, we studied how astrocytes shape signal formation and patterning during development and in non-perturbed conditions by analysing the differences in the recorded MEA signals. To study the astrocytes’ effects on the neuronal signals, we co-cultured rat primary cortical neurons and astrocytes in defined relative cell proportions; the co-cultures presented either 80% neurons and 20% astrocytes (referred to as ‘80/20 co-culture’) or 50% neurons and 50% astrocytes (‘50/50 co-culture’). As the control, we used cultures of rat cortical neurons with no explicitly added astrocytes (‘NS cultures’). To unravel signal differences between the NS and co-cultures, we applied traditional analysis like calculating the spike rates (SRs) and burst rates (BRs). Furthermore, to study how the astrocytes affected signal regularity, we assessed C-E planes for the signals of the different cultures at different developmental stages. To better describe the significance of the complexity and entropy parameters, we predicted the electrical signals of our MEAs using a random forest regressor [33,34]. Moreover, we classified the different spike waveforms present in our recordings and analysed several signal features such as amplitude, spike duration and baseline-to-spike slope. This study demonstrates the potential of using multidisciplinary approaches and applying analysis techniques more commonly used in other neuroscience applications to *in vitro* recordings to uncover new insights into signal control, formation and patterning by astrocytes. To the best of our knowledge, our study is the first that classifies the neuronal spike shapes and their repetitions in time with respect to the neuron–astrocyte ratios in the cultures to assess the role of astrocytes on ionic mechanisms. Furthermore, in our MEA signal analyses, we implemented complex systems analysis techniques commonly used in analysing EEG recordings. Through this in-depth signals analysis, we aim to shed light on how a decreased astrocytic ionic clearance can lead to increased neuronal hyperactivity and abnormal activity synchronization.

## 2. Methods

### 2.1. Cell culturing and plating

The day before cell plating, all sterilized MEAs (*n* = 18) and glass coverslips (*n* = 10) on a 24-well plate were coated with Poly-d-Lysine (0.1 mg ml^−1^; Thermo Fisher Scientific, Waltham, MA, USA) for 1 h at room temperature. The MEAs and the coverslips were washed three times with ultrapure water, air-dried in a laminar hood and incubated with laminin (L2020; 20 µg ml^−1^; Sigma-Aldrich, St Louis, MO, USA) overnight at +4°C. The following day, the MEAs and coverslips were taken to room temperature approximately 1 h before cell seeding, and laminin was aspirated just before the plating of neurons.

E18 primary rat cortex neurons (A1084001, Thermo Fisher Scientific) were thawed and counted using the Countess Automated Cell Counter (Thermo Fisher Scientific). After centrifuging cells (250*g* for 4 min), 80 000 neurons resuspended in 32 µl drops of medium were seeded onto each MEA and 40 000 neurons per coverslip. Neurons were left to attach in an incubator for 1 h. In the meantime, cryopreserved rat primary cortical astrocytes (N7745100, Thermo Fisher Scientific), previously treated with cytosine *b*-d-arabinofuranoside (ara-c, C1768, Sigma-Aldrich) [17], were thawed in their own medium, counted and centrifuged at 250*g* for 5 min. After the 1 h attachment time of the neurons had passed, 80 000 astrocytes in 40 µl drops of co-culture medium were seeded to yield the ratio of 50% neurons and 50% astrocytes (called ‘50/50 co-culture’) and 20 000 astrocytes in 10 µl drops of co-culture medium to yield the ratio of 80% neurons and 20% astrocytes (called ‘80/20 co-culture’) to the MEAs (*n* = 6 MEAs for each co-culture). Hence, the number of neurons always remained unchanged for all MEAs, and the number of astrocytes was adjusted for each co-culture condition. After seeding the ara-c-treated astrocytes, the MEAs with the co-cultures were further incubated for approximately 1 h before replenishing 1 ml of co-culture medium to each MEA. The number of astrocytes was also adjusted for the control coverslips (40 000 astrocytes for 50/50 and 10 000 for 80/20 co-cultures) and plated in a similar manner as the MEAs. For the neuron cultures without separately added astrocytes (called ‘NS culture’; six MEAs and six coverslips), after the first h of incubation, 1 ml of neurobasal plus medium was replenished per well and put to +37°C incubator in 5% CO${,}_{2}$ atmosphere. The neurobasal plus medium used for NS cultures contained neurobasal plus medium with 2% B-12 Plus supplement, 1% penicillin–streptomycin (P/S) and 0.25% GlutaMAX supplement. For the co-cultures, the medium was the same but included 1% GlutaMAX and 1% sodium pyruvate. The astrocyte medium used for the thawing comprised DMEM/F-12 (with HEPES, l-glutamine) with a 1% N-2 supplement, 1% P/S and 10% fetal bovine serum. All the products for different cell culture media were purchased from Thermo Fisher Scientific. At least half the volume of the cell culture medium was replaced three times a week, and always after the MEA recordings.

### 2.2. Immunostaining

Cell cultures (NS, 80/20 and 50/50) on coverslips were fixed, stained and mounted at 14 days *in vitro* (DIV) using the previously described protocol [17]. The used primary antibodies were Microtubule Associated Protein 2 (MAP2, chicken, PA1-10005, 1 : 1000) from Thermo Fisher Scientific and Glial Fibrillary Acidic Protein (GFAP, rabbit, AB5804, 1 : 1000) from Sigma-Aldrich. The secondary antibodies were anti-mouse Alexa fluor 488 (A-11001, 1:500), anti-rabbit Alexa fluor 555 (A-21428, 1 : 500) and anti-chicken Alexa fluor 647 (A32933, 1 : 500) (all from Thermo Fisher Scientific). All antibodies were diluted with 5% (v/v) goat serum in PBS purchased from Sigma–Aldrich. After washing the coverslips, 1 : 1000 4,6-diamidino-2-phenylindole (DAPI, 10 µg ml^−1^ in PBS, Thermo Fisher Scientific) was added for 10–15 min. The coverslips were stored at +4°C until imaging. Olympus IX51 Fluorescence Microscope with an Olympus DP30BW camera (Olympus Corporation, Hamburg, Germany) was used for imaging and Fiji (ImageJ) software for image processing. Images were further analysed with an in-house MATLAB (MathWorks, Inc., Natick, MA, USA) script [17], which can be found in GitHub at https://github.com/barbara-ge/NeuronAstrocyteCounter.

### 2.3. Microelectrode array recordings and preprocessing

In this study, we used standard 60-electrode MEAs (60MEA200/30iR; Multi Channel Systems MCS GmbH, Reutlingen, Germany). The raw signals were recorded two to three times a week for 5 min using a MEA2100 system and the Multi Channel Experimenter software (both MCS). Before recording, the MEAs were left on the recording stage for 5 min to settle. The recording sampling rate was 25 kHz. The cultures were analysed at 14, 19 and 28 DIV. All recordings were maintained at +37°C. Subsequently, the raw signals were filtered with a second-order bandpass elliptic filter in the range of 300–3000 Hz. Positive and negative spikes were detected when they exceeded the threshold of ±5σ, where σ is the standard deviation of the filtered signal. The threshold of ±5σ was chosen to ensure correct discrimination between noise and signal. Astrocytes seemed to slightly reduce the signal-to-noise ratio. Compared with the NS cultures, which showed distinct spikes above the baseline level, the co-cultures exhibited relatively lower number of such spikes. Thus, using a smaller sigma resulted in many false spike detections.

### 2.4. Spike and burst analysis

The filtered signals were further analysed with a MATLAB tool [35] that uses a network-wide cumulative moving average (CMA) algorithm [36] for the detection of bursts. We calculated the SR (in Hz), BR (in Hz), burst duration (BD; in ms), the percentage of spikes contained in bursts (% spikes in burst), the ISIs (in ms) and the IBIs (in ms). The aforementioned analyses were conducted in a time window of 100 s, between 100 and 200 s of the total recording time, to match the analysis time window used in the following analysis of the entropy and complexity of the signals.

### 2.5. Entropy and complexity analysis

We used an information-theoretic approach to analyse the MEA signals. Specifically, we used C-E causality planes [28,29,32] that employ the Bandt & Pompe approach [37] to define two information-theoretic metrics, the normalized Shannon entropic measure $H_{s}[P]$ [27], which characterizes the degree of regularity or orderliness of the signal, and the statistical complexity measure $C_{js}[P]$, which characterizes the physical structure of the signal [26,30].

Given a single-channel time series $X=\{x_{t}{\}}_{t=1}^{T}$, based on the approach by Bandt & Pompe [37], we can define ordinal patterns of dimension M for each time point u as follows:


(2.1) $$(u)\to (x_{u-(M-1)\tau },x_{u-(M-2)\tau },\ldots ,x_{u-\tau },x_{s}).$$

The M-dimensional vector $\left(x_{u-(M-1)\tau },x_{u-(M-2)\tau },\ldots ,x_{u-\tau )},x_{u}\right)$ assigned to each time u is obtained using Taken’s embedding theorem, and qualitatively represents the embedding vector at time point u for a given lag τ and dimension M. A sequence of integers $\left(s_{0},s_{1},\ldots ,s_{M-1}\right)$ is defined such that it describes the rank order of the components of the embedding vector (with 0 indicating the smallest value) and is a unique permutation of the set {0,1,…,M−1} satisfying [29]


(2.2) $$x_{u-s_{\left(M-1\right)}\tau }\leq x_{u-s_{\left(M-2\right)}\tau }\leq \ldots x_{u-s_{1}\tau }\leq x_{u-s_{0}\tau }$$

and


(2.3) $$s_{l}<s_{l-1}\;\text{if}\;x_{u-s_{l-1}}=x_{u-s_{l}}.$$

Note that there exists M! different possible ordinal patterns when a time series is embedded in M dimensions, and we denote these patterns by $\pi_{1},\pi_{2},\ldots ,\pi_{M!}$.

For all possible M! permutations, the associated relative frequencies for pattern $\pi_{i}$ can be computed as [29]


(2.4) $$p(\pi_{i})=\frac{\#\{u|u\leq T-(M-1)\tau ;(u)\;\text{has type}\;\pi_{i}\}}{T-(M-1)\tau },$$

where # stands for the number of occurrences. We can now define the normalized entropy measure $H_{s}[P]$ as


(2.5) $$\begin{array}{ccc} & H_{s}[P]=\frac{S[P]}{\;\log\;M!} & \end{array}$$

with


(2.6) $$S[P]=-\sum_{i=1}^{M!}p(\pi_{i})\log p(\pi_{i}),$$

and the complexity measure $C_{js}[P]$ as [29]


(2.7) $$\begin{array}{ccc} & C_{js}[P]=Q[P,P_{e}]H_{s}[P], & \end{array}$$

where $P_{e}$ represents the uniform probability distribution, i.e. $P_{e}=\{1/M,\ldots ,1/M\}$ and $Q[P,P_{e}]$ represents the disequilibrium in terms of the Jensen–Shannon divergence $J_{e}$ and is given as


(2.8) $$\begin{array}{ccc} & Q[P,P_{e}]=Q_{0}J_{e} & \end{array}$$

with


(2.9) $$J_{e}=S[(P+P_{e})]-S[P]/2-S[P_{e}]/2,$$

where S[⋅] is the Shannon entropy measure defined in equation (2.6).

For the C-E analysis, we used a time window of 100 s, between 100 and 200 s of the total recording time, as mentioned above. Furthermore, we used an embedding dimension 𝑀=5 and optimal time lag τ=100 samples. The signal was low-pass filtered at 40 Hz to denoise the signal and then down-sampled to 1 kHz. After preprocessing the signals, we obtained the ordinal pattern distributions used to estimate entropy and complexity as given in equations (2.5) and (2.7).

We used the iterative amplitude-adjusted Fourier transform (iAAFT) scheme [38], which employs an iterative approach to produce surrogates that have the same power spectra and values as the original data at hand to generate surrogates. The surrogates, which are chaotic by definition, will be used to test the null hypothesis that the underlying dynamics of the time series is a stationary, linear, stochastic and correlated process that is measured by a static, monotonic and possibly nonlinear observation function [38,39]. For each single-channel signal of the MEAs, we generated 49 iAAFT surrogates and then applied the same preprocessing and C-E analysis. From both the 60 original signals for each MEA, and the 2940 generated iAAFT surrogates (49 iAAFT surrogates for 60 channels), we measured the complexity and entropy and we generated the empirical cumulative distribution function (empirical CDF) of the measures. The empirical CDF is a step function that shows for any point of the series its probability of observation. The empirical CDFs generated from the complexity and entropy measures in the iAAFT surrogates of all the MEAs of each culture were then treated as ‘control’ in the statistical analysis. The used script is publicly available at https://github.com/narayanps/NolinearTimeSeriesAnalysis.

### 2.6. Waveform analysis

**Figure 1.**
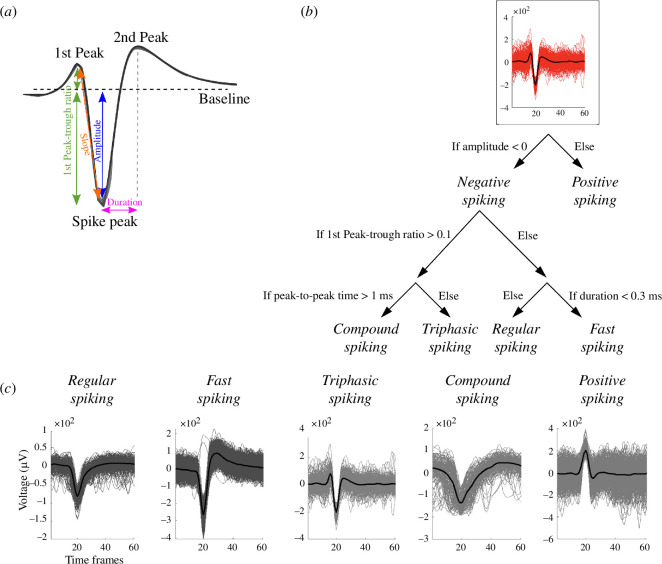
Waveform selection and features. (*a*) Waveform features used for the waveform analysis (modified from [41]) and (*b*) the decision tree of the algorithm used for the waveform detection. (*c*) Examples of the five possible wave shapes: RS, FS, TS, CS and PS.

The action potential spike cut-outs, i.e. waveforms, were extracted and sorted with a software tool called Wave_clus for MATLAB (R2009b or higher) [40]. The sorting algorithm differentiates all spike waveforms found in the recordings and clusters them based on the similarity of the waveforms. The waveforms were saved in time windows of a total of 3 ms (60 amplitude samples) with 1 ms before and 2 ms after a spike. All the spike waveforms sorted in the same cluster were then averaged (figure 1*b*, black line) to obtain the average waveform for each cluster. Then each averaged waveform was classified into five possible wave shapes: regular spiking (RS), fast spiking (FS), triphasic spiking (TS), compound spiking (CS) and positive spiking (PS) (figure 1*c*). We defined the waveforms based on the previous work from [41]. The features used to categorize and analyse the waveforms (figure 1*a*) were: the spike amplitude (µV), calculated as the amplitude between the baseline and the peak amplitude; the 1st peak-through ratio: the absolute value of the ratio between the amplitude of the 1st peak before the spike and the actual spike peak amplitude; the amplitude slope: (spike peak amplitude − 1st peak amplitude)/(spike time point − 1st peak time point); the peak-to-peak time (ms): the time between the 1st peak before the spike and the 1st peak after the spike (defined as 2nd peak); the spike duration (ms): the time between the spike peak and the 2nd peak. The baseline was calculated as the average amplitude between the first and the last timeframes. A representative image of the waveform features is shown in figure 1*a*.

For the waveform categorization, first of all, we categorized a waveform to be of the PS wave shape, if the spike amplitude was positive, otherwise, the spike was categorized as a negative spike. Subsequently, all the negative spikes were then further categorized based on the following rules:


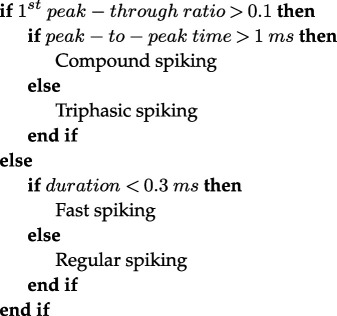


The decision tree for the waveform selection is shown in figure 1*b*, and the MATLAB code used for the waveform analysis is available on GitHub at https://github.com/barbara-ge/WaveformAnalysis.

### 2.7. Signal prediction with random forest

As confirmation of the meaning of the entropy and complexity measures on the signal predictability, we used a random forest regressor [33,34]. A random forest is an estimator that fits a set of classification decision trees to different subsamples of a dataset, using averaging to improve prediction accuracy and control overfitting. We used the algorithm implementation from the scikit-learn library [42] in Python 3. A preprocess of the raw signal data was conducted to decrease the data points in the time series and retain only import information. The signal was low-pass filtered with a Butterworth filter of 5th order and a low-cut frequency of 300 Hz, to remove as much noise as possible from the signals, without losing spikes. Moreover, the signals were down-sampled from 25 kHz to 250 Hz, meaning one timestamp for each 4 ms was maintained. This value was decided as a compromise between the spike duration we obtained from the signal analysis and the need to reduce the time points. For the random forest regressor, we did not impose any limitation on the maximum number of trees that the algorithm could create. The 5 min time series was split with a 70–30 train–test rule, such as the first 70% of the data points were used for training and the remaining 30% for testing. The regressor was run for all 59 channels (excluding the reference electrode) of all the MEAs, in each of the culture conditions (i.e. NS, 80/20 and 50/50). The model accuracy was then verified with a Pearson’s correlation analysis [43] between the predicted values and the real values in the test set. The implementation of the signal prediction model can be found on GitHub at: https://github.com/barbara-ge/MEA_signal_predictor.

### 2.8. Statistical analysis

Statistical analysis was conducted either in MATLAB or GraphPad Prism (v. 9; GraphPad Software, San Diego, CA, USA). To compare the different cultures, we used ordinary one-way ANOVA, and the test was considered significant for p< 0.05, where p is the probability of obtaining results in the tail of the distribution. The empirical CDF generated from the iAAFT surrogates and from the original signals were compared to test their distribution differences with a two-sample Kolmogorov–Smirnov test. The test rejects the null hypothesis, h=1, of having the data from the same distribution if p< 0.05; otherwise, h=0. Pearson’s correlation analysis was conducted in Python 3 with the SciPy library [44]. The correlation value *R*${,}^{2}$ can have values from −1 to 1. Values of −1 or +1 imply an exact linear relationship, while 0 implies no correlation [43].

## 3. Results

**Figure 2.**
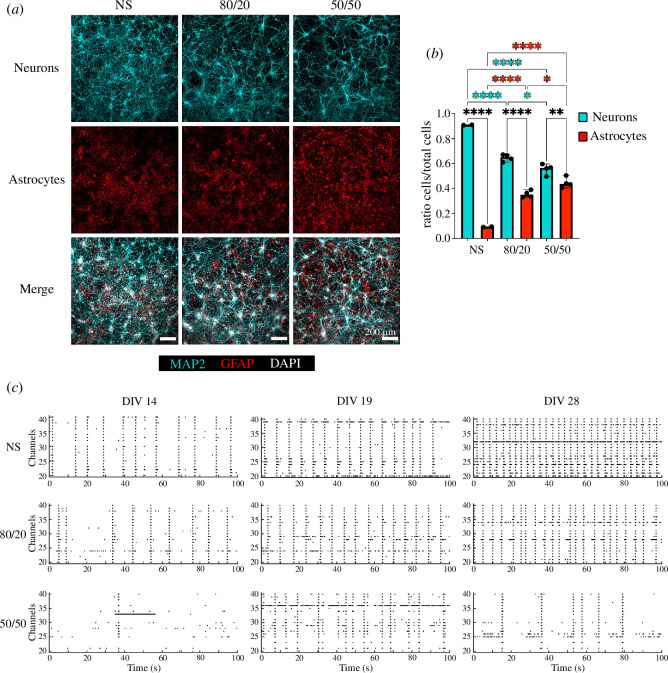
Immunostaining of neurons and astrocytes on coverslips. (*a*) In the first row MAP2 is represented in cyan for the NS, 80/20 and 50/50 cultures. In the second row, GFAP is shown in red and in the third row the merge of MAP2, GFAP and DAPI images of the same ROI for each culture is presented. The scale bar is 200 µm. (*b*) The relative cell counts of all ROIs and all coverslips are presented. The bars represent the percentage of neurons or astrocytes over the total number of DAPI-labelled nuclei. One-way ANOVA test results for the comparisons between neuron and astrocyte amounts in the culture are shown in black; the differences between the different cultures are shown in cyan for the neurons and red for the astrocytes. ^∗^<0.05; ^∗∗^<0.01; ^∗∗∗∗^<0.0001. (*c*) Representative raster plots of the measurements from the same MEAs at 14, 19 and 28 DIV, respectively. The figure represents the spiking activity of a snippet of 20 channels (rows) in the 100 s time window; the spike times are represented by black dots.

To verify the network development at DIV14, we immunostained the coverslips for the NS, 80/20 and 50/50 cultures with MAP2, GFAP and DAPI (4′,6-diamidino2-phenylindole). Figure 2*a* shows a representative image of one region of interest (ROI) of each culture; in the first row the neuronal network (MAP2), in the second row the astrocytes (GFAP) and in the third row the merge of the neuronal and astrocytic networks. Figure 2*a,b* illustrate that while the neuronal densities were very similar in all cultures, the number of astrocytes increased towards the 50/50 co-culture as intended. Figure 2*b* presents the result of the cell counting from coverslips of all the cultures. The bars represent the percentage of the specific type of cell over the number of total cells. NS presented an average of 90% neurons and 10% astrocytes, already existing in the commercially obtained neuronal cell stock. The 80/20 co-culture had 65% neurons and 35% astrocytes, and 55% neurons and 45% astrocytes for the 50/50 were found. The differences between the different cultures were all statistically significant and the obtained ratios well reflected the ratios of astrocytes plated.

### 3.1. Activity development

**Figure 3.**
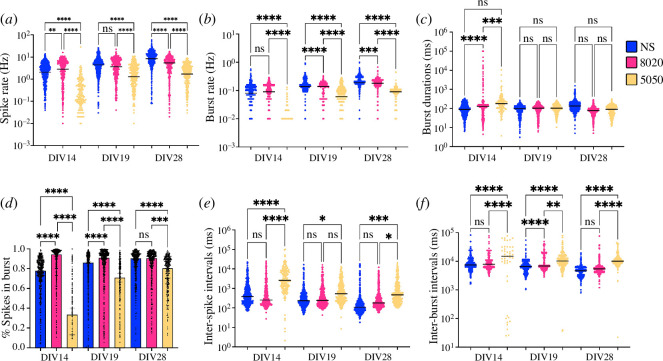
Activity development analysis. (*a*) SR (Hz), (*b*) BR (Hz), (*c*) BD (ms), (*d*) % spikes in bursts, (*e*) ISIs (ms) and (*f*) IBIs (ms) for NS (in blue), 80/20 (in magenta) and 50/50 (in yellow) cultures for 14, 19 and 28 DIV. The black bars in the violin plots represent the median. For the 50/50 co-culture in (*a*) and (*b*), the median and some of the points are hidden in the logarithmic scale towards the 0 value. One-way ANOVA test: ns, non-significant;.^∗^<0.05; ^∗∗^<0.01; ^∗∗∗^<0.001; ^∗∗∗∗^<0.0001.

To analyse the activity development, we calculated the SR and BR at 14, 19 and 28 DIV. The SR and BR developed and increased over the recording weeks (figure 3*a,b*). NS showed higher SR levels compared with 80/20 and 50/50 cultures starting from 19 DIV. At 14 DIV, the SR for NS was lower than for 80/20 but higher than for 50/50; no clear tendency was noticeable. From 19 DIV, a lower SR was noticeable for the co-cultures and almost linearly with the number of astrocytes in the culture. The BR followed a similar trend, respectively. At 14 DIV, the BD was longer for co-cultures with more astrocytes (figure 3*c*). At 19 and 28 DIV, even though the BD seemed lower for co-cultures and especially for the 50/50 co-cultures, the differences between NS, 80/20 and 50/50 cultures were not statistically significant (figure 3*c*). To assess network synchronization, we calculated the percentage of spikes contained in bursts of the total amount of spikes in the recording analysis window. There was an increase in synchronization over the recording weeks for the NS and 50/50 but not for the 80/20 cultures. The spikes were already almost all contained in bursts at 14 DIV (figure 3*d*). The 50/50 co-cultures showed lower synchronization compared with the other two. The ISIs decreased over the DIV recordings in general, and the 50/50 co-cultures showed higher ISIs compared with NS and 80/20 cultures (figure 3*e*). Similar behaviour was noticeable for the IBIs as well (figure 3*f*).

### 3.2. Signal patterning

Complexity and entropy planes have been used to study the signal structure and patterning of the single channels of the MEAs. At all DIV, the NS cultures presented higher complexity levels and lower entropy levels, followed by the 80/20 and then the 50/50 co-cultures.

**Figure 4.**
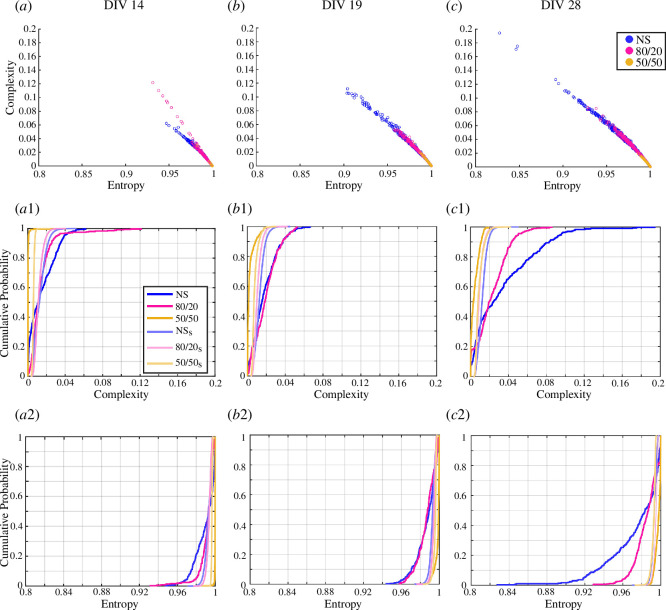
C-E planes and iAAFT surrogates analysis. (*a–c*) C-E values of the single channels in the cultures at DIV14, DIV19 and DIV28, respectively. NS cultures are shown in blue, 80/20 in magenta and 50/50 in yellow. The values for 50/50 were all clustered in the lower right corner, at high entropy and low complexity values, for all the DIVs tested. (*a1–c1*) Empirical CDF of the complexity values of the cultures on each measurement DIV, compared with the empirical CDF of the complexity levels of the relative generated surrogates (subscript s in the labels). The NS_s_ are shown in light blue, the 80/20_s_ in pink and the 50/50_s_ in light yellow. (*a2–c2*) Empirical CDF of the entropy measures of the cultures on each DIV, compared with the empirical CDF of the entropies of the relative generated iAAFT surrogates. The original complexity and entropy values from the C-E planes, and the entropy and complexity empirical CDFs were compared at each DIV with a two-sample Kolmogorov–Smirnov test.

Observing figure 4*a–c* reveals a clear trend in which the points representing the single channels of the MEAs shift towards lower levels of entropy and higher levels of complexity as the DIV increases, indicating a progressive increase in signal patterning. In contrast, the 50/50 co-cultures consistently exhibit high entropy and low complexity values, indicating a nearly unpatterned and random signal throughout all the DIV.

The empirical CDF of the complexity and entropy measures from the generated iAAFT surrogates at DIV14 presented fewer differences from those of the original signals (figure 4*a*1,*a*2). Between DIV19 and DIV28, the empirical CDFs of the complexity measure for the original signals in the NS cultures and 80/20 co-cultures shifted towards higher values. This indicates a greater likelihood of finding higher complexity values and thus a more patterned signal (figure 4*b*1*,c*1). In contrast, the empirical CDF of the entropy measures for the original signals in the NS cultures and 80/20 co-cultures showed an increased likelihood of lower entropy values (figure 4*b*2*,c*2). The NS cultures exhibited more pronounced changes in the complexity and entropy distributions than the 80/20 co-cultures, indicating a higher degree of signal patterning, as also demonstrated by the entropy–complexity planes (figure 4*b,c*). The empirical CDFs for both the complexity and entropy measures in the original signals of the 50/50 co-cultures consistently presented higher probabilities at lower values than those from the iAAFT surrogates, indicating a complete lack of signal patterning (figure 4*a*1–*c*2 ).

The empirical CDFs were proven different between those obtained from the measures from the original signals and the iAAFT surrogates in all cultures and at all DIV with the use of a two-sample Kolmogorov–Smirnov test. The single comparisons between the empirical CDF of the measures from the original signals and the generated iAAFT surrogates for all the cultures at each DIV are shown for more clarity in electronic supplementary material, figure S1 for the complexity and figure S2 for the entropy.

### 3.3. Waveform analysis

**Figure 5.**
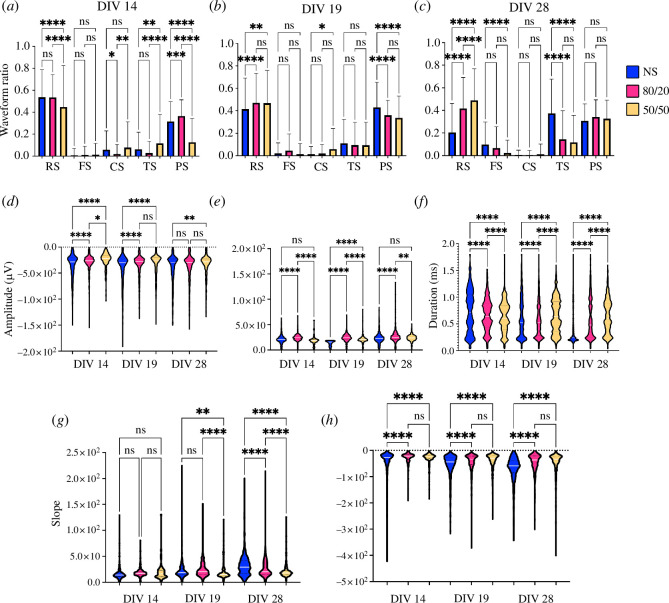
Waveform analysis. (*a–c*) Prevalence of the spike waveforms in the NS, 80/20 and 50/50 cultures (blue, magenta and yellow) at 14, 19 and 28 DIV, respectively. RS, regular spiking; FS, fast spiking; CS, compound spiking; TS, triphasic spiking; and PS, positive spiking. (*d*,*e*) Spike amplitude in µV at 14, 19 and 28 DIV for the negative and positive spikes, respectively. (*f*) Spike duration in ms, counted as the time from the spike peak to the 2nd peak (or to the end of the time window in the absence of the 2nd peak). (*g,h*) Positive and negative slopes, respectively, to reach the spike peak for the NS, 80/20 and 50/50 cultures at 14, 19 and 28 DIV. The white bars in the violin plots represent the median. One-way ANOVA test: ns, non-significant; ^∗^<0.05; ^∗∗^<0.01; ^∗∗∗^<0.001; ^∗∗∗∗^<0.0001.

The waveform analysis conducted on the spike cut-outs saved from the Wave_clus tool showed a prevalence of RS in all our co-cultures. At 14 DIV, NS and 80/20 cultures presented more RS than the 50/50, which presented more CS and TS instead, compared with NS and 80/20 cultures (figure 5*a*). At 19 DIV, the trend in RS switched and the 50/50 co-cultures showed a prevalence compared with the others (figure 5*b*); a trend that was then clear at 28 DIV (figure 5*c*). CS remained more prevalent in 50/50 compared with the other cultures at 19 DIV and 28 DIV, even though the differences were not statistically significant (figure 5*b,c*). However, CS was rare in general on all DIVs. PS prevalence increased from 14 DIV to 28 DIV for the 50/50 co-cultures but remained mostly constant for NS and 80/20. At 28 DIV, there were no statistically significant differences for the different cultures for the PS prevalence. FS and TS followed an inverse trend compared with RS; at 14 DIV, their numbers were slightly higher for the co-cultures and then reversed at 28 DIV. This trend is the consequence of the shift in spike duration as we can notice from 14 DIV to 28 DIV (figure 5*f*). The spike duration in fact remained mostly constant for the 50/50 co-cultures but shifted from longer duration to shorter most noticeably for the NS cultures and for the 80/20 co-cultures.

The differences in the spike duration between the co-cultures were also comparable to the differences in the positive and negative slopes, such as how fast the negative or positive spike peak, respectively, was reached from the 1st peak. At 14 DIV, the differences in the positive slopes were not statistically significant. As the DIV progressed, the slopes demonstrated an increase for the NS and 80/20 cultures, while a decrease was observed for the 50/50 co-cultures (figure 5*g*). This resulted in a faster reach of the spike peak for the NS and 80/20 co-cultures, and a slower reach for the 50/50 co-culture. The trend was similar also for the negative spikes; however, the differences between the two co-culture ratios, 80/20 and 50/50, were not statistically significant (figure 5*h*).

The spike amplitude of the negative spikes seemed to be lower for the 80/20 and 50/50 co-cultures on 14 and 19 DIV, but then at 28 DIV all amplitudes were similar (figure 5*d*). The amplitude of the positive spikes remained more or less constant throughout all DIV (figure 5*e*).

### 3.4. Signal prediction

**Figure 6.**
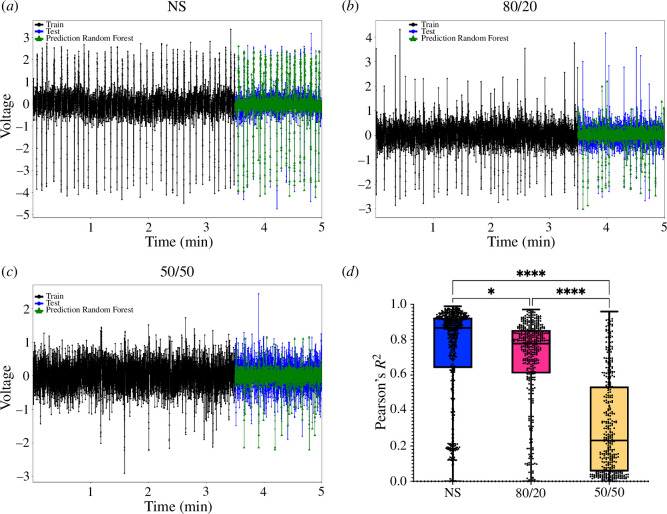
Random forest prediction. (*a*–*c*) Signals with the training set in black, the test set in blue and the random forest prediction in green, for NS, 80/20 and 50/50 cultures, respectively. (*d*) Pearson’s correlation analysis between the real values and the predicted values for all channels from all MEAs for NS (in blue), 80/20 (in magenta) and 50/50 cultures (in yellow). The black lines represent the medians and the whiskers show the minimum and maximum points of the datasets. One-way ANOVA test: ^∗^<0.05; .^∗∗∗∗^<0.0001.

Representative plots of the signal predictions in NS, 80/20 and 50/50 are displayed in figure 6*a–c*. Here, the black parts represent the training sets, the blue part represents the test set and green part represents the predicted signal. We noticed that in the co-cultures, where less repetitive spikes were present, the algorithm more often mispredicted the spikes. The cumulative results of all the predictions from all MEAs are represented in figure 6*d* in the form of boxplots of the Pearson’s *R^2^* correlation values between the predicted values and the relative real value. Thereby, the NS cultures had the highest accuracy with an *R^2^* value of 0.73 ± 0.28, the 80/20 co-cultures had an *R*^2^ value of 0.69 ± 0.24 and the 50/50 co-cultures had an *R*^2^ value of 0.31 ± 0.27. The black lines in the boxplots represent the median values of *R^2^*, which were 0.87 for NS, 0.78 for 80/20 and 0.23 for 50/50 cultures. In electronic supplementary material, figure S3, the comparison between all the predicted points and their relative test values for NS, 80/20 and 50/50 cultures is displayed.

## 4. Discussion

We investigated the astrocytes’ effects on the spiking and the shape of action potentials recorded with MEAs, as well as on signal development and patterning through the use of complex measures. We classified the shapes of the spike signals cut-outs to analyse the features in the different culture conditions; no added astrocytes, 20% added astrocytes and 50% added astrocytes, referred to as NS, 80/20 and 50/50 cultures, respectively. The co-cultures were obtained by first seeding the neurons and later adding the astrocytes to the cultures. We used entropy and complexity analysis techniques on the raw signals to deeply analyse the recorded signals and their patterning over time.

Studying the activity in co-cultures with fixed shares of cells is crucial to deeply understanding the seizure susceptibility of the different brain areas presenting different glial–neuron ratios (GNRs) [45]. The varying GNR across the brain areas seems to be rather constant between species, and it seems to reflect more a variability of neuronal density, rather than glial density [46]. However, for the sake of comparability between recorded activity in our MEAs, we decided to keep the neuronal density fixed and to adapt the astrocytic densities to have a linear increase of GNR from supposedly 0 in the NS to 1 : 1 in the 50/50 co-culture.

Our results showed that the decrease in SR and BR was larger for the 50/50 co-cultures compared with the 80/20 co-cultures at 28 DIV, suggesting that these directly depend on the number of astrocytes in the culture. Even though the total SR and BR were lower for the co-cultures, the 50/50 co-cultures showed a relatively higher change in SR and BR when comparing 19 and 28 DIV to 14 DIV, indicating a facilitated activity development in cultures with astrocyte support. Moreover, we showed that spiking and bursting appeared more prominently in the 80/20 co-culture at 14 DIV compared with NS. Additionally, the BD was longer for the co-cultures. Previous studies demonstrated that astrocytes support network development as well as increase the survival for long-term cell culture maintenance [47]. Furthermore, reduced spiking activity and a stabilization of the neuronal bursting activity were noticeable in matured human pluripotent stem cells co-cultures [48]; the prolonged differentiation of these co-cultures resulted in a higher presence of astrocytes, with an astrocyte presence ranging from 2% with eight weeks of differentiation to almost 50% with 15 weeks of differentiation, which resemble our co-cultures.

Astrocytes also changed the properties of the bursts; the percentage of spikes in bursts was lower for the 50/50 co-cultures than for NS and 80/20 cultures. These aforementioned results are in accord with a previous study we conducted [17], as well as with a computational study modelling neuron–astrocyte communication in networks [49]. Also, the IBIs increased for the 50/50 co-cultures together with its inner variability. Moreover, our results suggest that astrocytes not only limit high SRs and BRs but also decrease the synchronicity and the patterning of the signals that can be associated with epileptic-like behaviour [32].

Neural activity patterns in EEG signals have been more commonly analysed with complexity and entropy measures [50–52]. Multi-scale entropy, in particular, has been used to discriminate between different neurological disorders, and between patients with generalized seizures from those with focal seizures [50]. However, information measures alone are limited to discriminating random activity with certain neural activity due to the abstract approach in associating brain structure with activity [53]. Our C-E analysis showed that the complexity of the signals increased more prominently in the NS cultures during development. This measure indicates patterning and, thus, a more repetitive and synchronous activity. The co-cultures, instead, presented less repetitive and patterned activity in general when developed. However, the 80/20 cultures presented more patterning already at 14 DIV compared with NS.

The entropy measure discriminates stochastic activity with low entropy levels from chaotic activity with high entropy levels. Our results showed that entropy decreased during cell culture development. The NS cultures showed less chaotic behaviours, meaning lower entropy, compared with co-cultures. While high entropy values indicate a random activity of the network, it does not mean that said activity is truly random. Entropy as a measure is not enough to distinguish true randomness from an activity pattern that appears to be random. More studies can help clarify if the co-cultures present a protection mechanism to entropy variations as well as altering neural codes found *in vivo*. Interestingly, a recent study on classifiers to discriminate EEG data from epileptic patients and seizure-free patients found that the Shannon entropy was lower in epileptic patients compared with seizure-free individuals [51]. This suggests that an increase in overall neuronal activity with over-synchronicity can be well characterized by information measures with real impact on signal processing tools for diagnostics. Thus understanding real entropy variations considering information/complexity measures can produce predictors of neural states, associating areas of the brain with their structural configuration. As further confirmation of the biological meaning of the entropy and complexity measures, we used ML techniques to predict the voltage traces from the MEA electrodes under the three culture conditions. Considering the meaning of entropy, such as a description of the stochasticity of a signal, better prediction performances should be achieved at lower levels of entropy. To predict our signals, we used a random forest regressor [33,34], which is fast in computing and normally applied to reduce overfitting. In our data, NS signals were predicted quite accurately, whereas the 80/20 and 50/50 co-cultures instead presented a decreasing accuracy. These results confirm that signals with higher entropy and lower complexity are more difficult to predict. The higher accuracy for the NS cultures also probably depended on the higher number of spikes, and, thus, the algorithm can probably better discriminate between spikes and noise. An even better accuracy could be achieved by testing different ML algorithms for the regression or with more testing on the data preprocessing to further reduce the noise. However, since the prediction of the signals was not the main point of this study, we did not focus on improving the model performances.

To deepen the understanding of how astrocytes affect signal formation in neurons, we analysed the waveforms of the recorded spikes. The analysis methods used were similar to [41]. In [41], the authors used recorded signals from a cat’s visual cortex. The differences they observed in the spike waveforms were dependent on the type of neuronal populations in the different visual cortex areas from which they recorded.

In our investigation, our analysis of waveforms revealed that co-cultures exhibited a more consistent pattern of spiking and a reduced occurrence of FS compared with NS cultures. This was accompanied by a slower reach of the spike’s peak. This phenomenon is indicated by the smaller slope values and an extended interval between the spike’s peak and the subsequent peak. We believe that the decline in spike slope and the extension of spike duration in co-cultures can be attributed directly to the astrocytes’ role in clearing excessive ions from the extracellular space. The channels responsible for K^+^ clearance are the Na^+^/K^+^-ATPase, Kir4.1 and the Na^+^/K^+^/2 Cl${,}^{-}$ cotransporter (NKCC) [54–56]. During an action potential, astrocytes uptake both negative and positive charges through the NKCC and release positive charges through the Na^+^/K^+^-ATPase [54], resulting in a delayed shift in the relative charge between the intracellular and extracellular spaces. We believe that the presence of a greater number of astrocytes in proximity to the neurons contributes to the delayed spike peak, as demonstrated in our slope analysis findings. In the repolarization phase, extruded K^+^ from the neurons is taken up by the Na^+^/K^+^-ATPase and Kir4.1 in the astrocytes [56], causing a slower accumulation of positive charge in the extracellular space. This accounts for the prolonged duration between the spike peak and the subsequent peak observed in our results. Neurons are unable to initiate new spikes during the refractory period, encompassing depolarization, repolarization and part of the hyperpolarization phase [13]. The longer the neuron remains within these phases, the fewer spikes are initiated. In our study, co-cultures exhibited extended depolarization and repolarization phases in comparison with NS cultures, resulting in reduced spike frequencies in the co-cultures.

To the best of our knowledge, no other studies have compared neuronal cultures with co-cultures with fixed shares of astrocytes. Our study is the first one to deeply study how astrocytes modify the burst characteristics *in vitro* and highlight how astrocytes control the spiking activity of the neurons. Moreover, these results could be an insight into how the astrocytes prevent hyperactivity, and why epileptic seizures rise in animal models when the astrocytic K^+^ clearance is defective.

## 5. Conclusion

It is known that astrocytes play a crucial role in the development, support and maintenance of the CNS [2], and in the regulation of the neuronal activity, but the exact mechanisms behind this control are yet to be fully explored. However, it is known that dysfunctions in the astrocytes may lead to hyperactivity. In this work, we utilized analyses and metrics that up to now have not been commonly used in MEA recordings analysis, but are mostly used in information theory and signal processing of EEG recordings. By adopting uncommon analysis techniques we were able to study how action potential shapes and patterning were affected by the relative number of astrocytes in co-cultures. We showed that astrocytes modify how the action potential is formed in neurons, changing the relative spike features. Astrocytes decreased the signal slope and increased the spike duration, such as slowing both the depolarization and repolarization phases. The longer time spent by the neurons in the refractory phase resulted in lower SRs in co-cultures. Moreover, astrocytes increased chaotic behaviours in the recorded signals, decreasing the patterning at the single-channel level and leading to a less accurate prediction of the signals. At the network level, the signals presented more singular and sparse spikes and thus a lower percentage of spikes in bursts compared with the cultures of neurons without explicitly added astrocytes. With this work, we wanted to stress the importance of adopting interdisciplinary approaches and going beyond standard spike and burst analyses to deepen the knowledge of the astrocytic processes that regulate neuronal signalling. Also, we consider it to be of crucial importance to utilize fixed amounts of added astrocytes to define and control the relative neuron/astrocyte ratios in the plated co-cultures. In this study, primary rat neurons and astrocytes were used; however, it is well known that human astrocytes present unique morphological and functional differences compared with their rat counterparts [57]. Furthermore, recent works have shown that human-induced pluripotent stem cell (hiPSC)-derived astrocytes compare favourably with rat astrocytes [58], and the robustness of MEA-derived neuronal activity patterns from hiPSC has been benchmarked [22]. Hence, as a future extension of this study, it would be interesting to study the functions of human-derived astrocytes and their modulatory effects on neuronal networks, in healthy and pathological conditions, and whether the contributions of human astrocytes to neuronal functionality is, for example, more or less pronounced compared with rat models. In *in vitro* cultures, astrocytes improve the overall culture viability and stabilize the neuronal network, protecting it from drug-induced synapse loss [59] or drug-induced hyperactivity [17]; therefore, studying the entropy and complexity of hiPSC neuron–astrocyte co-cultures will give insights into how astrocytes affect and regulate the signalling cascades controlling the neuronal activity, and how they change under pharmacological modulation, thus leading to important insights to better understand neurodegenerative diseases and drive drug development.

## Data Availability

The raw data of part of the recordings at DIV14, DIV19 and DIV28 used in the current paper is publicly available at Zenodo [60]. Supplementary material is available online [61].
